# Effect of pH levels on platelet aggregation and coagulation: a whole blood *in vitro *study

**DOI:** 10.1186/cc9866

**Published:** 2011-03-11

**Authors:** G Scharbert, G Franta, L Wetzel, S Kozek-Langenecker

**Affiliations:** 1 Medical University Vienna, Austria; 2 Evangelical Hospital, Vienna, Austria

## Introduction

The combination of acidosis, hypothermia and coagulo-pathy is associated with high mortality in polytrauma [1]. Acidosis impairs coagulation [2]. Whether acidosis leads to a reduced platelet function has not so far been evaluated.

## Methods

In this *in vitro *study we evaluated the effects of pH levels (7.6, 7.4, 7.2, 7.0 and 6.8) on platelet aggregation and coagulation with human whole blood of healthy male volunteers. We used multiple electrode aggregometry (MEA) Multiplate^® ^(tests: ADP, ASPI, TRAP) for platelet function testing. The global coagulation was evaluated at pH 6.8 and 7.4 with ROTEM^®^, which is a rotational thrombelastometry (tests: NATEM and APTEM). The pH levels of the blood samples were achieved by titration of HCl and NaOH.

## Results

In MEA the AUC was significantly reduced for pH 7.0 and pH 6.8 in all three tests (ADP, ASPI and TRAP), as well as aggregation and velocity. Platelet function was not influenced by alkalosis (pH 7.6). In ROTEM^® ^the AUC, CT, CFT and MCF showed no significant alterations. The α-angle and lysis index for 60 minutes were significantly reduced at pH 6.8. NATEM values were significantly different from those measured with APTEM.

## Conclusions

In our study we evaluated a significant decrease of platelet function at pH 7.0 and 6.8 with MEA. The results of the analysis with the ROTEM^® ^system showed a significant reduction of thrombus formation at pH 6.8, as described in the literature. In the APTEM test, we could identify hyperfibrinolysis.
